# mir-34b/c and mir-449a/b/c are required for spermatogenesis, but not for the first cleavage division in mice

**DOI:** 10.1242/bio.201410959

**Published:** 2015-01-23

**Authors:** Shuiqiao Yuan, Chong Tang, Ying Zhang, Jingwen Wu, Jianqiang Bao, Huili Zheng, Chen Xu, Wei Yan

**Affiliations:** 1 Department of Physiology and Cell Biology, University of Nevada School of Medicine, Reno, NV 89557, USA; 2 Department of Histology and Embryology, Shanghai Jiao Tong University School of Medicine, Shanghai 200025, China; 3 Shanghai Key Laboratory of Reproductive Medicine, Shanghai 200025, China

**Keywords:** Epigenetics, Spermatogenesis, Fertility, Germ cell, Reproduction

## Abstract

Mammalian sperm are carriers of not only the paternal genome, but also the paternal epigenome in the forms of DNA methylation, retained histones and noncoding RNAs. Although paternal DNA methylation and histone retention sites have been correlated with protein-coding genes that are critical for preimplantation embryonic development, physiological evidence of an essential role of these epigenetic marks in fertilization and early development remains lacking. Two miRNA clusters consisting of five miRNAs (miR-34b/c and miR-449a/b/c) are present in sperm, but absent in oocytes, and miR-34c has been reported to be essential for the first cleavage division *in vitro*. Here, we show that both *miR-34b/c*- and *miR-449*-null male mice displayed normal fertility, and that intracytoplasmic injection of either *miR-34b/c*- or *miR-449*-null sperm led to normal fertilization, normal preimplantation development and normal birth rate. However, *miR-34b/c* and *miR-449* double knockout (miR-dKO) males were infertile due to severe spermatogenic disruptions and oligo-astheno-teratozoospermia. Injection of miR-dKO sperm into wild-type oocytes led to a block at the two-pronucleus to zygote transition, whereas normal preimplantation development and healthy pups were obtained through injection of miR-dKO round spermatids. Our data demonstrate that miR-34b/c and miR-449a/b/c are essential for normal spermatogenesis and male fertility, but their presence in sperm is dispensable for fertilization and preimplantation development.

## INTRODUCTION

Once inside the oocyte, the sperm head, with the paternal genome heavily packed inside, starts to de-condense and forms the paternal pronucleus (Yanagimachi, 2003; Yanagimachi, 2005a; Yanagimachi, 2012). Therefore, sperm have long been regarded as a vehicle for paternal genome delivery. Research over the past two decades has revealed that sperm deliver not only the paternal genome, but also factors required for oocyte activation, first cleavage division and the subsequent preimplantation development (Sutovsky, 2011; Jenkins and Carrell, 2012a; Yanagimachi, 2012). In addition to protein factors, sperm have been found to carry coding and noncoding RNA species into oocytes during fertilization (Lalancette et al., 2008; Jodar et al., 2013). Moreover, some sperm DNA methylation patterns appear to be preserved after post-fertilization reprograming, and loci associated with retained histones have been correlated with genes critical for early development (Hammoud et al., 2009; Brykczynska et al., 2010; Smith et al., 2012). Numerous intact mRNAs have been detected in sperm (Ostermeier et al., 2004; Martins and Krawetz, 2005; Jodar et al., 2013). Given the lack of functional translation machinery, these sperm-borne mRNAs must be for the post-fertilization usage if they do have a role. Recent deep sequencing of human and mouse sperm have revealed numerous noncoding RNA species, including miRNAs, piRNAs, tRNA-derived small RNAs, rRNA-derived small RNAs and snoRNAs (Lalancette et al., 2008; Peng et al., 2012; Jodar et al., 2013; Sendler et al., 2013). Small noncoding RNAs have been shown to affect mRNA stability and translational efficiency at post-transcriptional levels; or alternatively, to function as epigenetic factors in controlling gene expression at transcriptional levels (Saxe and Lin, 2011). These recent discoveries strongly suggest that sperm may contribute, in addition to genetic codes, epigenetic information during fertilization (Jenkins and Carrell, 2012b; Gannon et al., 2014).

While physiological evidence supporting essential roles of specific sperm DNA methylation and histone retention patterns in preimplantation development remains lacking, a recent study reports that injection of a “miR-34c inhibitor” into zygotes attenuated the first cleavage division after fertilization, and based on this finding, it was concluded that a single sperm-borne miRNA, miR-34c, has an essential role in the first cleavage division (Liu et al., 2012). However, the validity of this *in vitro* finding needs to be confirmed using *in vivo* mouse models in which sperm are devoid of miR-34c. Moreover, miR-34c belongs to a family of five miRNAs including miR-34b, miR-34c, miR-449a, miR-449b, and miR-449c, which are encoded by two miRNA gene clusters: miR-34b/c and miR-449. All five miRNAs have the same “seed” sequence and thus, target the same sets of mRNAs (He et al., 2007; Choi et al., 2011; Marcet et al., 2011; Bao et al., 2012). Given that the LNA-based miRNA inhibitors are usually designed to block the seed sequences of miRNAs, it is possible that the “miR-34c inhibitor” used in the previous report (Liu et al., 2012) might have inhibited all members of this miRNA family, thus leading to the phenotype reported. To evaluate whether miR-34c and the other 4 members of the miRNA family have an essential role in the first cleavage division both *in vivo* and *in vitro*, we analyzed *miR-34b/c* (Choi et al., 2011) and *miR-449* (Bao et al., 2012) knockout mice, and also generated *miR-34b/c; miR-449* double knockout (herein called miR-dKO) mice. By natural mating, intracytoplasmic sperm injection (ICSI), and round spermatid injection (ROSI), we report, here, that although these five miRNAs are indeed expressed in sperm and absent in oocytes, they are dispensable for fertilization, oocyte activation, first cleavage division and all subsequent steps of preimplantation development. Interestingly, these five miRNAs are required for normal spermatogenesis and male fertility.

## RESULTS

### miR-34b/c and miR-449a/b/c are expressed in sperm, but absent in oocytes

**Fig. 1.**
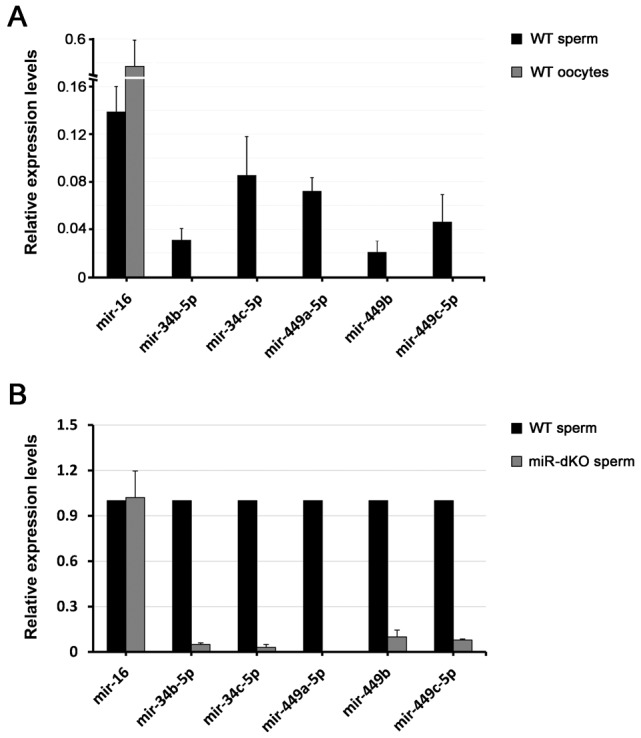
Expression of the five miRNAs encoded by two miRNA clusters in mouse sperm and oocytes. (A) qPCR analyses of levels of miR-16 (positive control), miR-34b/c and miR-449a/b/c in wild-type (WT) mouse sperm and oocytes. Data are presented as mean±SEM (n = 3). (B) qPCR analyses of levels of miR-16 (positive control), miR-34b/c and miR-449a/b/c in wild-type (WT) and *miR-34b/c;miR-449* double knockout (miR-dKO) sperm. Data are presented as mean±SEM (n = 3).

Using TaqMan-based miRNA qPCR analyses, we examined expression levels of miRNA-34b/c and miR-449a/b/c in mouse sperm and oocytes (Fig. 1A). Consistent with the earlier report, all five miRNAs were only detected in sperm, but completely absent in oocytes (Liu et al., 2012). Although miR-dKO males are infertile, a small number of sperm could still be recovered from the dKO epididymides, as described below. Using qPCR, we analyzed levels of the five miRNAs in WT and miR-dKO sperm (Fig. 1B). Consistent with a complete inactivation of the two miRNA clusters, levels of all five miRNAs were undetectable in dKO sperm (Fig. 1B).

### Inactivation of either the *miRNA-34b/c* or the *miR-449* miRNA cluster does not affect fertility

**Fig. 2.**
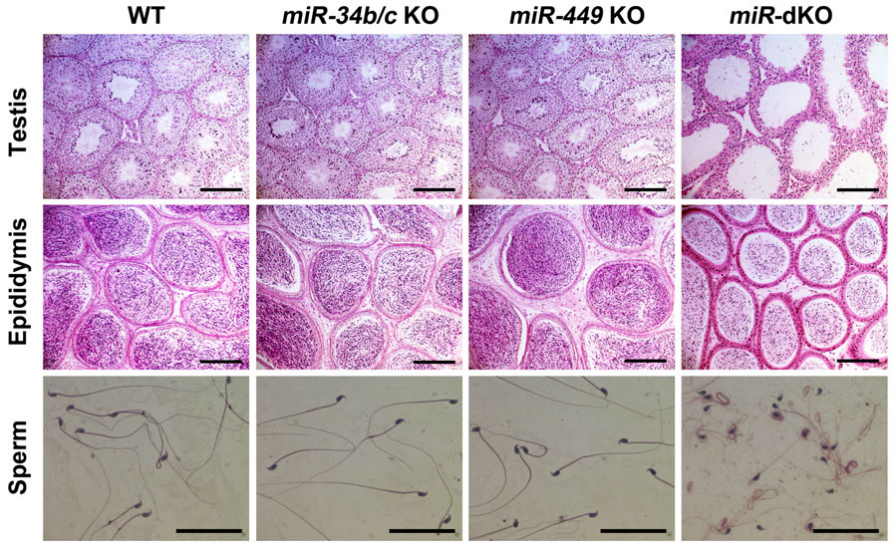
Testicular and epididymal histology and sperm morphology of wild-type (WT), *miR-34b/c* knockout (KO), *miR-449* KO, and *miR-34b/c;miR-449* double KO (miR-dKO) male mice at the age of 10 weeks. Note that the miR-dKO testes displayed thinner seminiferous epithelia and larger lumens, as compared to WT control and single KO testes, and the histology of miR-dKO testes is similar to that reported recently (Wu et al., 2014). Scale bars = 200 µm (upper and middle panels); 50 µm (lower panels).

**Fig. 3.**
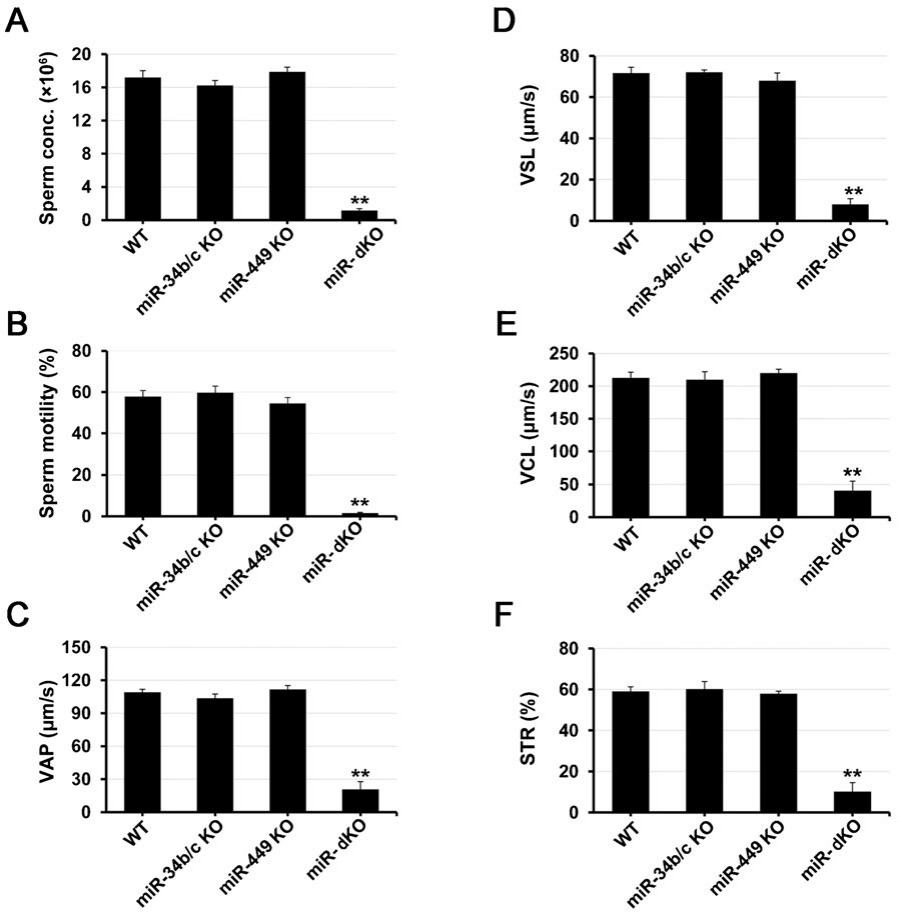
Computer-assisted sperm analyses (CASA) of epididymal sperm collected from wild-type (WT), *miR-34b/c* knockout (KO), *miR-449* KO, and *miR-34b/c;miR-449* double KO (miR-dKO) male mice. Parameters analyzed included sperm count (A), total motility (B), average path velocity (VAP) (C), straight line velocity (VSL) (D), curvilinear velocity (VCL) (E), and straightness (STR = VSL/VAP) (F). Data are presented as mean±SEM (n = 6). **P<0.01.

As reported previously, global *miR-34b/c* KO and *miR-449 KO* mice are viable (Choi et al., 2011; Bao et al., 2012). To evaluate their fertility, adult *miR-34b/c* KO males were mated with either adult WT or miR-34b/c KO females. Similarly, adult miR-449 KO males were bred with adult WT or miR-449 KO females. Litter number, size, and interval were recorded (supplementary material Table S1). We observed no differences between WT controls and mating pairs with different combinations between KO and WT mice, suggesting that both *miR-34b/c* and *miR-449* global KO males and females both have normal fertility. Consistent with the normal male fertility, we observed normal testicular histology (Fig. 2), normal sperm morphology (Fig. 2), and normal sperm counts and motility (Fig. 3). Therefore, inactivation of either of the two miRNA clusters individually does not affect male fertility.

### Both *miR-34b/c-* and *miR449-*null sperm can fertilize wild type oocytes and support embryonic development

To test if *miR-34b/c*- and *miR-449*-null sperm can fertilize WT oocytes and support preimplantation development, we performed ICSI using epididymal sperm isolated from these two KO males. As described above, these miRNA KO sperm lacked expression of either miR-34b/c or miR-449 (Fig. 1B). By injecting *miR-34b/c*-null sperm into WT or *miR-34b/c*-null oocytes, we evaluated the potential of preimplantation development from 2-pronuclear (2PN) to blastocyst stages, and no significant differences were noted between WT control and the *miR-34b/c*-null sperm groups (supplementary material Table S2; Fig. S1), suggesting that the *miR-34b/c*-null sperm are competent for fertilization and the subsequent preimplantation development. Similar ICSI experiments were performed using *miR-449*-null sperm, and no effect on fertilization and preimplantation development was observed (supplementary material Table S3; Fig. S1). These negative data are consistent with the normal fertility test results (supplementary material Table S1), demonstrating that a lack of either *miR-34b/c* or *miR-449a/b/c* in sperm does not affect oocyte activation, first cleavage division, or subsequent preimplantation development both *in vivo* and *in vitro*.

### miR-dKO mice are infertile due to severe spermatogenic disruptions and oligo-astheno-teratozoospermia

All five miRNAs have the same “seed sequence” and thus, can target the same sets of mRNAs and be functionally redundant (Choi et al., 2011; Bao et al., 2012). Functional redundancy could explain the lack of discernable phenotype in single miRNA cluster KO males. Therefore, we and others generated miR-dKO mice by crossing these two KO lines (Comazzetto et al., 2014; Wu et al., 2014). Consistent with these two recent reports (Comazzetto et al., 2014; Wu et al., 2014), miR-dKO males were completely infertile after mating with WT females of proven fertility for 4 months (supplementary material Table S1). Moreover, the miR-dKO testes contained thinner seminiferous epithelia and larger lumens, as compared to WT control and single KO testes at 10 weeks of age (Fig. 2). Although all types of spermatogenic cells were present, the total cell number appeared to be drastically reduced in miR-dKO males (Fig. 2). A small number of seemingly fully developed sperm were present in the epididymis, but most of the miR-dKO sperm were deformed (Fig. 2). As demonstrated in our recent report (Wu et al., 2014), despite normal epididymal histology in miR-dKO males, up to 80% of the miR-dKO epididymal spermatozoa are deformed, and ∼67% of the deformed spermatozoa are headless. Computer-assisted sperm analyses (CASA) revealed significantly reduced sperm counts, minimal total motility and other motility defects (Fig. 3). The spermatogenic disruptions in miR-dKO male mice resemble oligo-astheno-teratozoospermia in men, and it is highly likely that this primary testicular failure leads to the complete infertility in miR-dKO males.

### miR-dKO sperm fail to activate WT oocytes and support further development after ICSI

To test whether sperm lacking all five functionally related miRNAs (miR34b/c and miR-449a/b/c) could fertilize WT oocytes and support early embryonic development, we performed ICSI using miR-dKO sperm and WT oocytes (Table 1). Interestingly, we observed significantly decreased developmental potential, even at the 2-PN stage, with only ∼28% of the oocytes injected with miR-dKO sperm reaching 2PN, compared to 91% when WT sperm were used (Table 1). A complete block was observed at the 2-cell to 4-cell transition (Table 1). To improve the post-ICSI activation rate, we performed artificial oocyte activation following ICSI using miR-dKO sperm (Kimura and Yanagimachi, 1995b), which yielded a slightly better outcome (Table 1). However, the developmental rates at the 2-PN and 2-cell stages remained significantly lower in the miR-dKO group, as compared to the WT control group (Table 1). These results suggest that miR-dKO sperm neither activate WT oocytes nor support the first cleavage division and the subsequent early embryonic development.

**Table 1.**
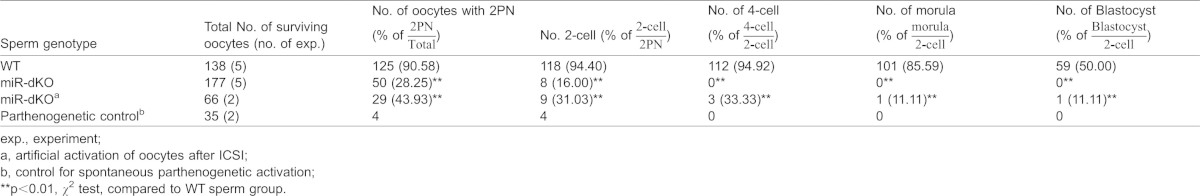
Fertilization and development of WT oocytes injected with WT or miR-dKO (*miR-34b/c*^−/−^;*miR-449^−^*^/−^) spermatozoa

### miR-dKO round spermatids can fertilize WT oocytes and support embryonic development

Given the severe spermiogenic defects in miR-dKO males, the inability of miR-dKO sperm to fertilize WT oocytes and support early development may well result from structural abnormalities due to aberrant spermiogenesis, which could include not only the lack of the five miRNAs, but also the absence of other essential factors or compromised integrity of the paternal genome. WT round spermatids can fertilize WT oocytes through ROSI, in spite of the fact that round spermatids have not developed the unique structures that are essential for oocyte activation and early developmental events (Yanagimachi, 2005a; Yanagimachi, 2005b). Therefore, a comparative study between miR-dKO and WT ROSI would allow us to unequivocally determine whether the five sperm-borne miRNAs are essential for the first cleavage division and the subsequent embryonic development, while eliminating other confounding variables resulting from aberrant spermiogenesis. Our results showed that when miR-dKO round spermatids were used in ROSI, the developmental potential was similar to that of WT ROSI (Table 2). We also transferred 2-cell embryos derived from miR-dKO ROSI and produced 4 living pups (supplementary material Fig. S2), which were all heterozygotes, suggesting they were truly derived from injected miR-dKO sperm. The ROSI pups developed normally and were indistinguishable from those derived from WT ROSI (Table 2). These results suggest that the inability of miR-dKO sperm to activate and support early development beyond the 2-PN stage is likely caused by indirect structural defects in the sperm rather than direct lack of the five sperm-borne miRNAs. Thus, all five sperm-borne miRNAs, including miR-34b/c and miR-449a/b/c, are dispensable for the first cleavage division and subsequent early embryonic development.

**Table 2.**
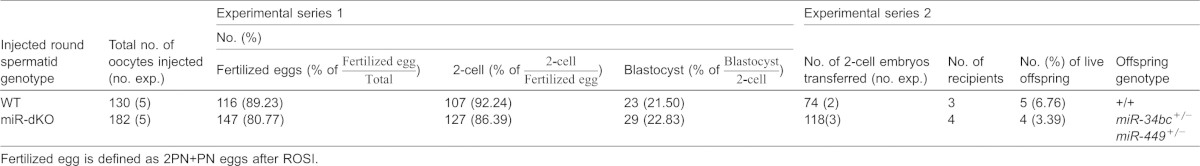
Term development of mouse embryos developed from the oocytes fertilized by injection of WT and miR-dKO (*miR-34b/c^−/−^; miR-449^−/−^*) round spermatids

### Disrupted spermiogenesis causes structural defects in miR-dKO sperm

**Fig. 4.**
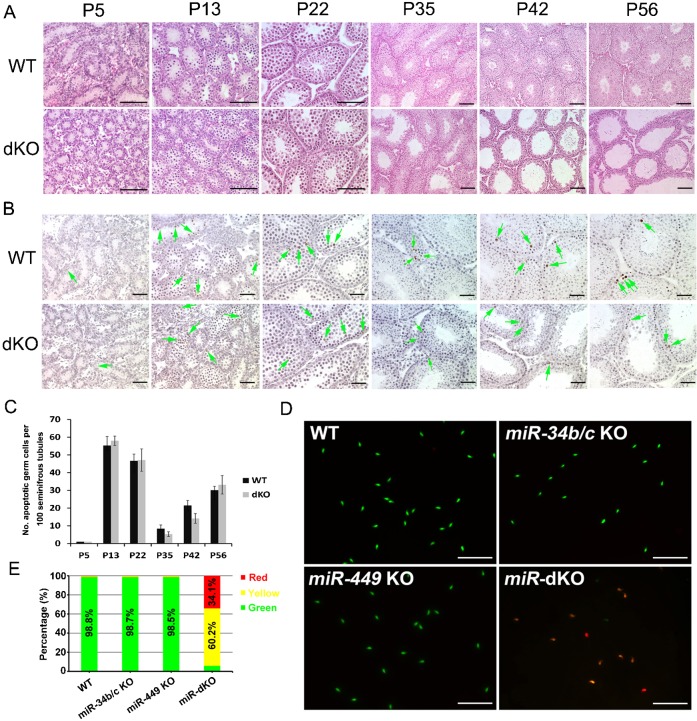
Histological and TUNEL analyses on developing testes of wild-type (WT) and *miR-34b/c;miR-449* double KO (miR-dKO) male mice and the acridine orange (AO) staining of WT and miR-dKO spermatozoa. (A) Representative HE-stained paraffin sections of developing testes at postnatal 5 (P5), P13, P22, P35, P42 and P56 from WT and miR-dKO male mice. Scale bars = 100 µm. (B) Representative results of TUNEL assays on paraffin sections of WT and miR-dKO developing testes at postnatal 5 (P5), P13, P22, P35, P42 and P56. The apoptotic cells are stained in brown (green arrows). Scale bars = 50 µm. (C) Quantitative analyses of apoptotic germ cells in developing WT and miR-dKO testes at P5, P13, P22, P35, P42 and P56. X-axis shows the age and the y-axis represents the total number of TUNEL-positive germ cells per 100 seminiferous tubule cross sections. Data are presented as mean±SEM (n = 3). No significant differences were observed. (D) Representative immunofluorescent images showing the results of AO staining on epididymal spermatozoa collected from WT, *miR-34b/*c KO, *miR-449* KO, and miR-dKO mice. Scale bars = 100 µm. (E) Quantitative analyses of AO staining results. Bars represent proportions of red, yellow/orange, or red sperm in WT, *miR-34b/*c KO, *miR-449* KO, and miR-dKO mice.

Histological analyses of the adult miR-dKO testes revealed severely disrupted spermatogenesis (Fig. 2). To determine the onset of spermatogenic disruptions, we further examined developing testes at postnatal day 5 (P5), P13, P22, P35, P42, and P56 (Fig. 4A). No obvious histological differences were noted between WT and miR-dKO testes at P5, P13, and P22. However, at P35, the seminiferous epithelia appeared to contain fewer germ cells and thus, looked thinner in miR-dKO testes compared to WT testes, and the thinner seminiferous epithelia became increasingly obvious from P42 to P56 (Fig. 4A).

To determine whether the thinner seminiferous epithelia in miR-dKO testes were caused by enhanced germ cell apoptosis, we performed TUNEL assays on WT and miR-dKO testes, and quantified the total number of TUNEL-positive germ cells per 100 randomly chosen tubule cross sections (Fig. 4B,C). Interestingly, no enhanced male germ cell apoptosis was observed although the miR-dKO seminiferous tubules did contain much fewer germ cells compared to WT controls, suggesting that either the overall efficiency of spermatogenesis in miR-dKO testes is compromised, or the spermatogenic cells are depleted through a non-apoptotic manner.

Failure of miR-dKO spermatozoa to fertilize WT oocytes in ICSI suggests that those mutant spermatozoa are functionally incompetent. To identify the causes of sperm dysfunction, we first conducted the acridine orange (AO) staining, also called spermatozoa chromatin structure assay (SCSA) (Venkatesh and Dada, 2010), which is based on the metachromatic shift from green (native chromatin) to red (denatured chromatin). The AO staining represents a sensitive structural probe for chromatin structure and packaging, and thus, has been widely used for assessing sperm chromatin maturity and quality (Fuse et al., 2006; Venkatesh and Dada, 2010). While >98% of the spermatozoa from WT or single KO males displayed green chromatin, only ∼6% of the miR-dKO sperm heads were stained green. ∼34% were red and the remaining ∼60% were yellow or orange (Fig. 4D,E). These data demonstrated poor chromatin quality in the miR-dKO sperm, which was likely caused by defective packaging during late spermiogenesis. Poor sperm chromatin is often associated with failure in fertilization and in supporting preimplantation development, which is consistent with what we observed in our ICSI study, as described above (Table 1).

**Fig. 5.**
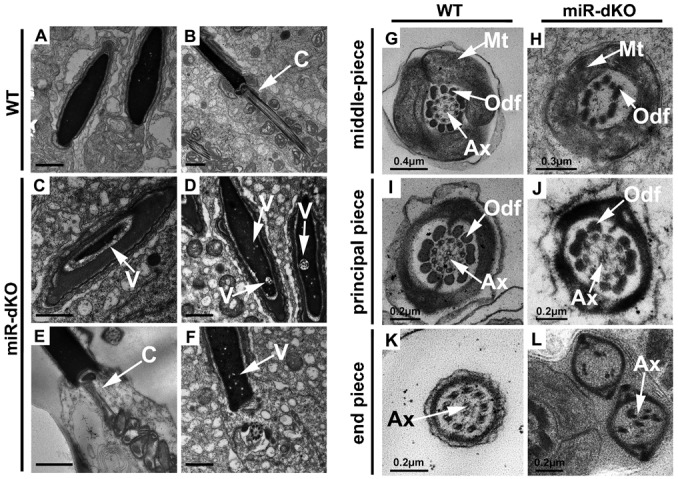
Chromatin condensation and flagellar defects in miR-dKO spermatozoa revealed by transmission electron microscopy (TEM). (A–F) Ultrastructral abnormalities in the chromatin and the connecting piece of miR-dKO spermatozoa. Unlike WT spermatozoa showing homogenously compacted chromatin (A,B), the chromatin in miR-dKO spermatozoa appeared to be heterogeneous with large (A) or small (D,F) vacuoles (“V”). The connecting piece (marked as “C”) of WT spermatozoa consists of segmented columns that are connected to the outer dense fibers (ODF, marked as “Odf”), whereas the miR-dKO spermatozoa often display atypical connecting piece with partial (E) or completely lacking (F) the segmented columns and the developing ODFs. Scale bars = 1 µm (A–F). (G–L) Flagellar defects in miR-dKO spermatozoa. In the middle piece, the mitochondrial sheath in WT spermatozoa is fully formed surrounding the ODFs and axoneme (G), whereas the mitochondrial sheath of miR-dKO spermatozoa is often partially formed and surrounds disorganized ODFs, enveloping partially formed or no axoneme (H). In the principal piece of WT spermatozoa, both 9 ODFs and 9+2 microtubules of the axoneme are well organized (I). In contrast, the miR-dKO spermatozoa display disorganized ODFs and atypical 9+2 microtubules of the axoneme (J) in the principal piece. In the end piece of WT spermatozoa, the axoneme with typical 9+2 microtubules is well organized (K), whereas the miR-dKO axoneme in the end piece displays disrupted microtubules without the typical 9+2 arrangement (L). Mt, mitochondrial sheath; Odf, outer dense fiber; Ax, Axoneme. Scales are labeled on panels G-L.

The oligo-astheno-teratozoospermic (OAT) phenotype in miR-dKO males strongly suggests spermiogenic defects (Yan, 2009). To reveal structural defects of miR-dKO spermatozoa, we further analyzed their ultrastructure using transmission electron microscopy (TEM) (Fig. 5). Almost all WT spermatozoa examined displayed homogenously condensed chromatin (Fig. 5A) and normal connecting pieces (Fig. 5B), whereas miR-dKO spermatozoa often showed chromatin lacking compaction (Fig. 5C), or containing large or small vacuoles (Fig. 5D,F), and partially formed, (Fig. 5E) or completely lacking (Fig. 5F) connecting pieces. TEM analyses of cross-sections of the sperm flagellum identified structural defects in axoneme, outer dense fiber (ODFs), and mitochondrial sheath along the flagella of miR-dKO spermatozoa (Fig. 5G–L). In the mid-piece, WT spermatozoa possess well-defined mitochondrial sheath enveloping 9 ODFs and axoneme, which consists of the typical “9+2” microtubules (Fig. 5G), whereas miR-dKO spermatozoa displayed disorganized mitochondrial sheath, ODFs, and axoneme microtubules, which, in some cases, were completely absent (Fig. 5H). In the principal piece, miR-dKO spermatozoa, unlike the WT controls (Fig. 5I), displayed mostly disorganized ODFs and perturbed “9+2” microtubular structures in the axoneme (Fig. 5J). Compared to the end piece in WT sperm (Fig. 5K), abnormalities in miR-dKO sperm end pieces became more obvious (Fig. 5L), completely lacking the typical organization and morphology of the “9+2” microtubular structures in the axoneme. These severe flagellar defects may explain why miR-dKO spermatozoa are mostly immotile (Fig. 3B). The flagellar defects in miR-dKO spermatozoa mostly reflect dysfunctions in centrioles because the connecting piece and the sperm flagellum start their development from the proximal and distal centrioles, respectively (Chemes and Rawe, 2010). Together, these functional and ultrastructural analyses indicate that the five miRNAs directly, or indirectly, affect sperm chromatin condensation and flagellogenesis during late spermiogenesis. Therefore, it is critical to identify the direct or indirect targets of the five miRNAs that are responsible for the spermiogenic defects in miR-dKO males, which are reported below.

### Altered expression of miRNA target genes is responsible for disrupted spermiogenesis in miR-dKO males

**Fig. 6.**
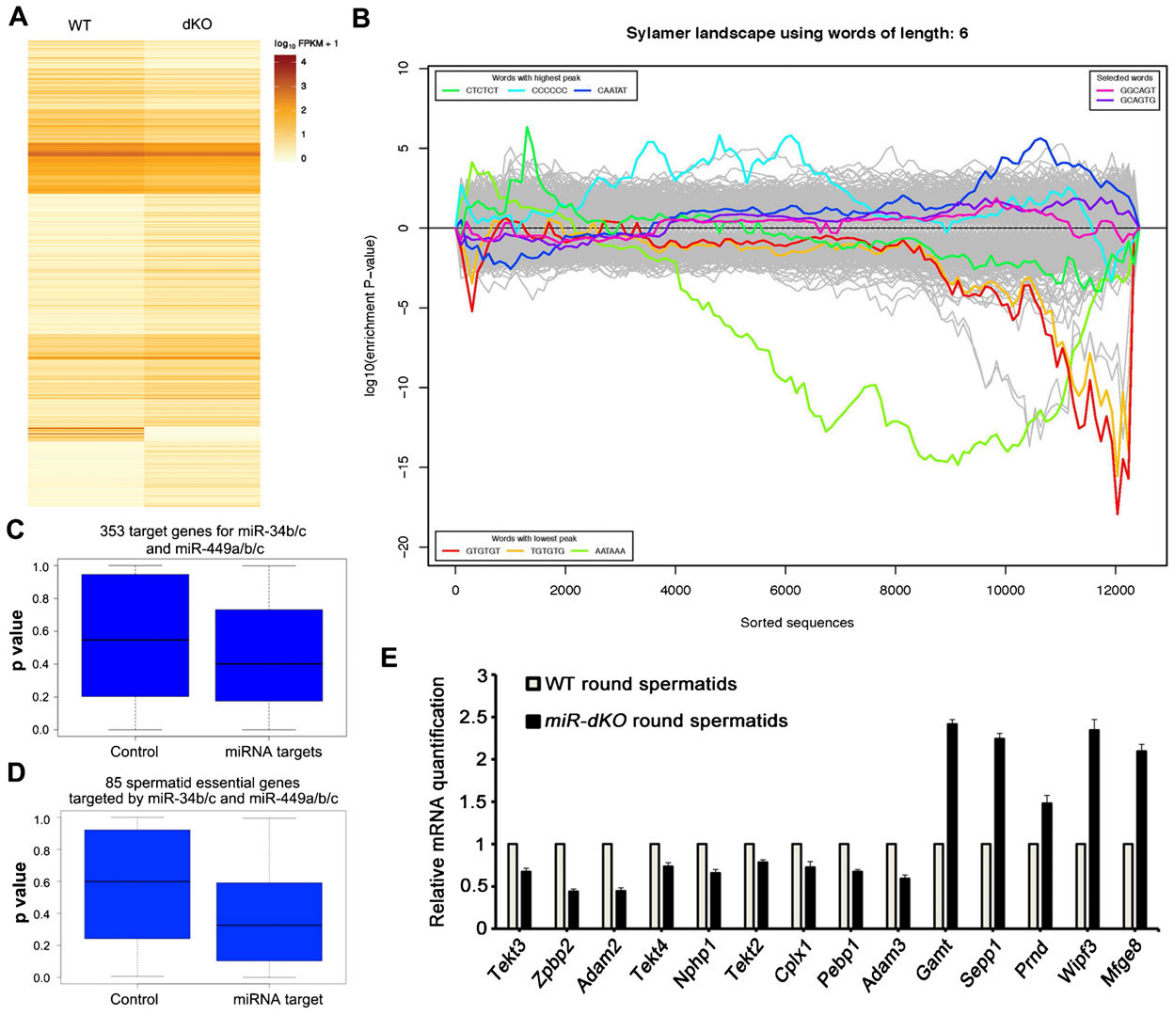
RNA-Seq analyses of the mRNA transcriptomes in WT and miR-dKO round spermatids. (A) Heatmap showing significantly dysregulated genes in miR-dKO round spermatids (compared to WT round spermatids). All 2,386 dysregulated genes are listed in supplementary material Table S4. (B) Sylamer analyses of the dysregulated mRNAs in miR-dKO round spermatids. Since the five miRNAs (miR-34b, miR-34c, miR-449a, miR-449b and miR-449c) share the same seed sequence of “GGCAGUG”, we analyzed two possible 6nt seed sequence combinations, including one with the 1^st^–6^th^ nt and the other with the 2^nd^–7^th^ nt (“selected words”). The most enriched 6nt sequences (“words with the highest peak”) were not the seed sequence of the five miRNAs, suggesting that the changes in the mRNA transcriptome are likely caused by secondary effects due to dysregulation of miRNA direct targets in dKO organs. (C) Boxplot of p values of miRNA target and control genes in the RNA-Seq data. To demonstrate that inactivation of the five miRNAs truly affects their target gene expression, the p values (dKO vs. WT) of all 353 miRNA target mRNAs and 353 randomly sampled, non-target mRNAs in the RNA-Seq data were plotted and compared. Paired t test was used and p = 2.351×10^−5^<0.05. (D) Boxplot of p values of miRNA targets known to be essential for late spermiogenesis and control genes in the RNA-Seq data. To demonstrate that inactivation of the five miRNAs truly affects their target genes known to be required for late spermiogenesis, the p values of all 85 miRNA target mRNAs and the same number of randomly sampled, non-target control mRNAs in the RNA-Seq data (dKO vs. WT) were plotted and compared. Paired t test was used and p = 5.473×10^−5^<0.05. (E) qPCR validation of 14 dysregulated mRNAs known to be required for late spermiogenesis. *Gapdh* was used as an internal control. Data are presented as mean±SEM (n = 3).

miRNAs function as post-transcriptional regulators, mainly by affecting the stability of their target mRNAs (Guo et al., 2010). To reveal mRNA transcriptomic changes responsible for the spermiogenic disruptions observed in miR-dKO males, we conducted RNA-Seq analyses using round spermatids purified from WT and miR-dKO testes at P56. During spermiogenesis, as soon as round spermatids start to elongate, transcriptional machinery is completely shut down due to the onset of chromatin condensation. Therefore, mRNAs needed for protein production in elongating and elongated spermatids are all transcribed in round spermatids and stored for later usage (Idler and Yan, 2012). Hence, round spermatids contain all the transcripts needed for the entire late spermiogenesis, which was why we chose round spermatids for RNA-Seq analyses. A total of 2,386 mRNAs were significantly dysregulated in miR-dKO round spermatids (negative binomial regression analysis, p<0.05), including 861 downregulated, and 1,525 upregulated mRNAs (Fig. 6A; supplementary material Table S4). Based on enrichment among all possible 6nt sequences in the 3′UTRs of dysregulated mRNAs, Slylamer analyses have been utilized to identify miRNA targets directly using the RNA-Seq data (van Dongen et al., 2008; Bartonicek and Enright, 2010). Although Sylamer analyses on our RNA-Seq data identified 3 highly enriched 6nt sequences, none matched the seed sequence of the five miRNAs (Fig. 6B), suggesting that the transcriptomic changes in miR-dKO round spermatids do not represent the primary effects of dysregulated direct targets for the five miRNAs.

Since Sylamer analyses failed to identify direct targets of the five miRNAs, we adopted TargetScan (Friedman et al., 2009; Garcia et al., 2011), and identified 353 target genes that were detected in both WT and miR-dKO round spermatids by RNA-Seq (supplementary material Table S5). Among the 353 target genes, 47 were significantly dysregulated, including 43 upregulated and only 4 downregulated (p<0.05, supplementary material Table S5). To evaluate whether these target genes, as a whole, were significantly affected in miR-dKO round spermatids, we randomly selected 353 non-target genes expressed in round spermatids and compared the p-values between the target and non-target genes in the RNA-Seq data (Fig. 6C). Interestingly, the average p-value of the 353 target genes was significantly lower than that of the control non-target genes, suggesting that the expression of these direct target genes is indeed altered in miR-dKO round spermatids.

Gene knockout studies over the past two decades have identified numerous genes essential for late spermiogenesis (Matzuk and Lamb, 2008). Among 109 late spermiogenesis-essential genes reported (Matzuk and Lamb, 2008), 85 were present in both WT and miR-dKO round spermatids (supplementary material Table S6). 15 out of the 85 late spermiogenesis-essential genes were significantly dysregulated in miR-dKO round spermatids, including 10 downregulated and 5 upregulated (supplementary material Table S6). Similarly, we compared the p-values of the 85 late spermiogenesis-essential genes with those of 89 randomly chosen non-target genes, and found that those spermiogenesis-essential genes were indeed affected (Fig. 6D). Among the 15 significantly dysregulated late spermiogenesis-essential genes, *Ros1*, *Nphp1* and *Sepp1* are known to be involved in chromatin condensation (Cooper et al., 2004; Olson et al., 2005; Jiang et al., 2008); and *Tektin2*, *3*, and *4* are required for sperm flagellogenesis (Matzuk and Lamb, 2008; Roy et al., 2009; Mariappa et al., 2010). 14 out of the 15 dysregulated late spermiogenesis-essential genes were also confirmed using qPCR analyses (Fig. 5E). Disruptions of these genes should be, at least in part, responsible for the defective chromatin condensation and flagellar formation observed in the dKO male mice. Together, these molecular analyses suggest that ablation of these five miRNAs causes transcriptomic changes due to the dysregulation of both target and non-target genes.

## DISCUSSION

The five miRNAs encoded by the two miRNA clusters, i.e., miR-34b/c and miR-449a/b/c, belong to the same miRNA family because they share the same “seed sequence” and target the same sets of mRNAs. Some of these mRNAs have been found to be mostly involved in the pRb-E2F1 cell cycle and the Bcl2 apoptotic pathways (Choi et al., 2011; Bao et al., 2012). miR-449 KO mice are viable and fertile (Bao et al., 2012), and here we show that miR-34b/c global KO mice also display normal fertility. Identical expression profiles between miR-34b/c and miR-449a/b/c, and the upregulation of miR-34b/c in miR-449 KO testes (Bao et al., 2012), strongly suggest that these two miRNA clusters might be functionally redundant. The fact that a lack of testicular phenotype when either of these two miRNA clusters is inactivated, while severe spermatogenic disruptions and male infertility in miR-dKO males, indicates that these two miRNA clusters are indeed functionally redundant. The onset of discernable disruptions in miR-dKO testes at P35 coincides with the beginning of sperm elongation and chromatin condensation, suggesting that late spermiogenesis is most severely affected. Disruptions in late spermiogenesis usually lead to decreased total sperm counts, increases in deformed and immotile spermatozoa, mimicking oligo-astheno-teratozoospermia in men (Yan, 2009).

Consistent with previous studies (Bao et al., 2012; Liu et al., 2012), our qPCR data further confirm that the five miRNAs are indeed present in spermatozoa, but absent in oocytes, and thus, represent paternal miRNAs. The earlier study claiming that miR-34c, as a paternal miRNA, is essential for the first cleavage division utilized a “miR-34c inhibitor” to suppress miR-34c function by injecting the inhibitor into zygotes (Liu et al., 2012). However, those *in vitro* findings were not validated using *in vivo* mouse models, e.g. using *miR-34c*-null spermatozoa for natural mating, IVF, and ICSI. Normal fertility of *miR-34c* or *miR-449* KO males suggests that sperm-borne miR-34c or miR-449 alone is dispensable for fertilization and early development. The fact that both *miR-34b/c*-null and *miR-449*-null spermatozoa perform as efficiently as the WT spermatozoa in ICSI demonstrates that a lack of either of the two miRNA clusters does not affect fertilization and early development either *in vitro* or *in vivo*.

Given the functional redundancy between the two miRNA clusters, it is not surprising to see no effect on fertilization and post-fertilization development when either is absent in spermatozoa. Although the miR-dKO sperm completely lack all five miRNAs, the caveats of using miR-dKO sperm for ICSI lies in that these sperm are mostly deformed due to disrupted spermiogenesis, and thus, may bear structural defects and compromised integrity of the paternal genome in addition to the lack of five sperm-borne RNAs. Therefore, the finding that miR-dKO sperm cannot activate WT oocytes and initiate the first cleavage division might result from factors other than the direct effects of lacking the five paternal miRNAs. Indeed, the sperm ultrastructral (via TEM analyses) and chromatin (through AO staining) analyses demonstrate that miR-dKO spermatozoa are defective in both the chromatin and the flagellum. The unstable chromatin in miR-dKO spermatozoa is likely the causes for the failure in fertilization, and in supporting post-fertilization development, that we observed in our ICSI assays. Therefore, we used ROSI to test whether miR-dKO round spermatids, which lack all five miRNAs, could support the first cleavage division and the rest of preimplantation development. Similar ROSI efficiency between WT and miR-dKO round spermatids and no block in the zygote to 2-cell transition, as well as the birth of healthy pups derived from ROSI using miR-dKO round spermatids, all support the notion that the five sperm-borne miRNAs are not required for the first cleavage division and subsequent development.

Because of the technical difficulties in generating gene knockouts, many choose to study gene functions using the knock down approach. It is, however, commonly known that mice that are heterozygous for most of the essential genes are phenotypically normal, suggesting that one needs to suppress the target gene expression/function by >50% in order to induce phenotypes. This could be challenging in practice if the RNAi or the “dominant negative” approach is used, where even with >50% suppression, one may not see the phenotype as clearly as in true knockouts with close to 100% suppression. The “miR-34c inhibitor” used in the previous study were LNA-based RNA oligos, designed to complimentarily anneal to the target miRNAs, thereby eliciting an effect through competitive inhibition (Liu et al., 2012). If the inhibitor used did in fact block the seed sequence of miR-34c, then all five miRNAs would have been suppressed as they all share this seed sequence. However, given that a total loss of all five paternal miRNAs has no effect on the first cleavage division based on our ICIS and ROSI data, the suppressive effects on the first cleavage division observed in that study (Liu et al., 2012) may imply some unknown “off-target” effects, which are common in miRNA inhibitor experiments *in vitro* (Ishida and Selaru, 2013). Therefore, it is imperative to validate *in vitro* miRNA inhibition data using data from *in vivo* miRNA KO models.

Although RNA-Seq analyses identified numerous dysregulated mRNAs, only ∼2% of the dysregulated genes are direct targets of the five miRNAs, suggesting that changes in the other 98% of identified mRNAs were most likely the representation of secondary effects. The failure of Sylamer analyses to identify direct targets of the five miRNAs also supports this notion. This is expected given that the five miRNAs are expressed in all earlier spermatogenic cell types including spermatogonia and spermatocytes (Bao et al., 2012). Aberrant gene expression may have been accumulated when they reach the haploid phase (i.e., in spermatids). Changes in both miRNA target genes and late spermiogenesis-essential, non-target genes in miR-dKO round spermatids strongly suggest that the spermiogenic disruptions result from the dysregulation of not only the target genes of the five miRNAs, but also non-target genes. Further molecular analyses are needed to discover the cascade of events that leads to defective chromatin condensation and flagellogenesis in the absence of the five miRNAs during later spermiogenesis.

In summary, although either of the two miRNA clusters (miR-34b/c and miR-449) is dispensable for male fertility, ablation of both results in disrupted spermatogenesis and male infertility. Despite their presence in sperm, miR-34b/c and miR-449a/b/c are not required for fertilization, first cleavage division, or subsequent development.

## MATERIALS AND METHODS

### Animals

All animal work was performed following the protocol approved by the Institutional Animal Care and Use Committee (IACUC) of the University of Nevada, Reno. Mice were housed and maintained under specific pathogen-free conditions with a temperature- and humidity-controlled animal facility in the University of Nevada, Reno. *miR-449* and *miR-34b/c* knockout mice were generated as described (Choi et al., 2011; Bao et al., 2012). All mice used in this study were on the C57BL/6J background.

### Chemicals and media

All chemicals were purchased from Sigma (St. Louis, MO) unless otherwise stated. For collecting sperm or oocytes, a modified CZB-HEPES medium containing 20 mM HEPES-Na, 5 mM NaHCO_3_, and 0.1 mg/ml polyvinyl alcohol (cold water soluble) was used. For culturing oocytes before ICSI or ROSI, a CZB medium supplemented with 5.56 mM D-glucose and 4 mg/ml BSA (Fraction V, Calbiochem, Temecula, CA) was used as described (Chatot et al., 1990; Kimura and Yanagimachi, 1995a; Yanagimachi et al., 2004). For culturing fertilized embryos after ICSI or ROSI, the KSOM medium (EmbryoMax®) supplemented with amino acids (KSOM+AA, Cat# MR-121-D, Millipore, Temecula, CA) was used.

### Preparation and collection of mouse oocytes

Adult (6–8 weeks) WT, *miR-449* KO and *miR-34b/c* KO female mice were superovulated using pregnant mare's serum gonadotropin (PMSG, 5 IU/mouse, i.p.), followed by human chorionic gonadotropin (hCG, 5 IU/mouse i.p.) 48 h later. Mature oocytes were collected from oviducts 14–16 h after hCG injection and cumulus cells were removed by treatment with 0.1% bovine testicular hyaluronidase in HEPES-CZB at 37°C for 2–3 min. Cumulus-free oocytes were used for either RNA extraction or ICSI and ROSI.

### Intracytoplasmic sperm injection (ICSI) and round spermatid injection (ROSI)

ICSI was performed as described (Kimura and Yanagimachi, 1995a; Stein and Schultz, 2010). Briefly, WT and KO sperm were collected in 500 µl HEPES-CZB medium and centrifuged at 700 g for 5 min. The sperm pellet was then resuspended in 200 µl NIM/PVA medium followed by sonication four times with 15 s each. An aliquot of 1–2 µl sperm suspension was mixed with 100 µl NIM/PVA medium (Stein and Schultz, 2010), and a single sperm head was pick up and injected into WT or KO oocytes using Piezo-driven manipulators (Eppendorf) under inverted microscope (Carl Zeiss) at RT. ROSI was performed as described (Kimura and Yanagimachi, 1995b) with slight modifications. Briefly, round spermatids were identified by size and morphology in WT or dKO testicular cell suspension. Individual round spermatids were drawn into an injection pipette, and through repeated aspiration, round spermatids were separated from each other and a single cell was injected into an oocyte through Piezo-driven micromanipulators. Prior to injection, oocytes were activated in Ca^2+^-free CZB medium containing 10 mM SrCl_2_ for 30–60 min at 37°C as described (Kimura and Yanagimachi, 1995b). Following microinjection, oocytes which survived from the injections were transferred into the KSOM+AA medium for incubation under 5% CO_2_ in humidified atmosphere at 37°C. Fertilization was confirmed 5–8 h post injection, and embryonic development following ICSI or ROSI was assessed every 24 h up to 5 days.

### Embryos transfer

Two-cell embryos (20–25) were transferred into the oviducts of a pseudopregnant CD1 female as described (Kimura and Yanagimachi, 1995b). Cesarean section was performed on day 19 after embryo transfer and pups were transferred to foster mothers.

### RNA isolation from mouse sperm and oocytes

Sperm small and total RNAs were isolated using the mirVana^TM^ miRNA isolation kit (Ambion, Grand Island, NY) as described (Yan et al., 2008; Wu et al., 2012). Sperm RNA samples were then stored at −80°C until qPCR analyses. Superovulated oocytes were randomly pooled into three groups with ten oocytes in each. RNA was released from oocytes in each group in 0.5 µl of 2 M guanidine isothiocyanate (Sigma) at RT for 5 min, and the samples were diluted to 5 µl with nuclease free water and directly used for qPCR analyses. Large RNA isolation from purified round spermatids was performed using the method described for RNA-Seq analyses (see below).

### Quantitative real-time PCR (qPCR)

miRNA qPCR analyses were carried out using the TaqMan miRNA assays as described (Wu et al., 2014). Sperm and oocyte small RNAs were reverse transcribed into cDNAs using the TaqMan miRNA reverse transcription kit (Applied Biosystems) according to the manufacture's instructions. qPCR was performed using the real-time PCR system (Applied Biosystems 7900). Conditions for PCR were 95°C for 10 min, followed by 40 cycles of 95°C for 15 sec and 60°C for 1 min. qPCR analyses of 14 miRNA targets known essential for late spermiogenesis were conducted as described (Song et al., 2011; Wu et al., 2012; Bao et al., 2014). U6 snRNA was used as miRNA qPCR data normalization. *Gapdh* was used as an internal control for qPCR validation of target mRNA genes. The primer sequences are listed in supplementary material Table S7.

### Sperm analysis

A computer-assisted sperm analysis (CASA) system (version 14.0, Hamilton-Thorne Bioscience, Beverly, MA, USA) was used to analyze sperm parameters including sperm concentration (million per ml), total motility (%), average path velocity (VAP, µm/s), progressive velocity (VSL, µm/s), curvilinear velocity (VCL, µm/s), straightness (STR, as VSL/VAP, %). WT and KO mouse sperm were collected from adult cauda epididymides and released into HTF medium, followed by incubation at 37°C for 1 h. Then, the sperm suspension was diluted to a proper concentration and 5 µl were loaded to a chamber slide (depth = 20 µm, Hamilton Thorne Research) for CASA.

### Histology analysis

For histological analyses, testes and epididymides were dissected from WT or KO mice. After fixation in Bouin's solution overnight at 4°C, the samples were embedded in paraffin blocks. Sections (5 µm) were cut and then stained with hematoxylin and eosin, or for the use of TUNEL assays. For sperm morphological analyses, cauda epididymal sperm from WT and KO mice were spread onto Superfrost Plus slides (Fisher Scientific, Hampton, NH) and air-dried. Sperm smears were stained with hematoxylin and eosin followed by microscopic examination.

### Acridine orange (AO) staining

WT and miR-dKO cauda epididymides were dissected and allowed for sperm release into the HTF medium at room temperature. Sperm suspensions were smeared onto Superfrost Plus slides (Fisher Scientific, Hampton, NH) followed by air dry and fixation in the Carnoy's solution (one part glacial acetic acid: three parts methanol) for 2 h. After fixation, slides were air-dried and stained with freshly prepared AO staining solution (0.19 mg/L, Polysciences, Warrington, PA, USA) for 5 min in the dark, as described (Chohan et al., 2004; Fuse et al., 2006). The stained slides were gently rinsed in distilled water, covered with glass cover slips and immediately evaluated under a fluorescent microscope at the excitation wavelength of 450–490 nm. Sperm displaying green fluorescence were considered with normal DNA content, whereas sperm emitting yellow–orange to red fluorescence were considered with damaged DNA. For each genotype, three mice were analyzed and >200 sperm cells were counted for each individual samples to quantify the percentage of spermatozoa with green, yellow/orange or red fluorescence.

### Transmission electron microscopy (TEM)

TEM was performed as described previously with some modifications (Yuan et al., 2013). Briefly, small pieces of WT and miR-dKO testes were fixed in 0.1 M cacodylate buffer (pH 7.4) containing 3% paraformaldehyde and 3% glutaraldehyde plus 0.2% picric acid for 2 h in 4°C, then for 1 h at RT. Following washes with 0.1 M cacodylate buffer, the samples were post-fixed with 1% OsO_4_ for 1 h at RT. Dehydration was performed using 30%, 50%, 70%, 90% and 100% ethanol solutions sequentially, followed by infiltration of propylene oxide and Eponate with BDMA overnight at RT. After infiltration, samples were embedded in Eponate mixture (Electron Microscopy Sciences, Hatfield, PA, USA) and polymerized at 60°C for 24 h. Ultrathin sections (60–70 nm in thickness) were cut with a diamond knife using an ultra-microtome (Leica). The sections were collected on collodion covered electron microscope nickel grids and stained with uranyl acetate and lead citrate. The ultrastructure of the samples was observed and photographed using a transmission electron microscope (Phillips CM10) at 80 kV.

### RNA-Seq analysis

Round spermatids were purified from WT and miR-dKO adult testes using a mini-STA-PUT method (Ro et al., 2007; Song et al., 2009; Wu et al., 2012). Large RNA was isolated from round spermatids using the mirVana RNA isolation kit (Ambion) according to the manufacturer's instructions. All samples are in biological triplicates. RNA quality and quantity were assessed using the Agilent 2100 Bioanalyzer. RNA-Seq was performed using an Illumina HiSeq 2000 sequencer (100 bp paired-end reads).

### Bioinformatic analysis

RNA-Seq data were processed using Tophat (Trapnell et al., 2009) and Cufflinks (Trapnell et al., 2010) following a published protocol (Trapnell et al., 2013). Target genes of the five miRNAs were determined using the Bioconductor Package-targetscan.Mm.eg.db [citation “targetscan.Mm.eg.db” (Krek et al., 2005)]. Sylamer analyses were conducted as described (van Dongen et al., 2008; Wu et al., 2014). In brief, the cuffdiff-processed RNA differential expression data were processed by using cutoff p-value≤0.05. The significantly dysregulated genes were arranged by the order of fold change, and UTR data were obtained through the bioconductor package “Genomicranges”. The arranged datasets were then processed using Sylamer and the settings were the same as those used in the *miR-155* knockout mouse study (van Dongen et al., 2008).

### Statistical analysis

For bioinformatic analyses, pipeline-specific statistical methods were used as described (van Dongen et al., 2008; Trapnell et al., 2009; Trapnell et al., 2010; Trapnell et al., 2013). Other data were shown as mean±SEM, and statistical differences between datasets were assessed by one-way ANOVA using the SPSS16.0 software. p≤0.05 was considered as significant differences, and p≤0.01 was considered as highly significant differences. ICSI data were analyzed using χ^2^ tests, and p≤0.05 was regarded as significant differences. χ^2^ tests were also used to compare the total number of pups born through ROSI. For miRNA qPCR analyses, the ΔCt method was used to calculate the relative miRNA expression levels in the experimental group and the control group.

### List of abbreviations

ICSI, intracytoplasmic sperm injection; dKO, double knockout; ROSI, round spermatid injection; 2-pronucleus, 2-PN; TUNEL, terminal deoxynucleotidyl transferase dUTP nick end labeling; TEM, transmission electron microscopy.

## Supplementary Material

Supplementary Material
