# Supplementary material for: mir-34b/c and mir-449a/b/c are required for spermatogenesis, but not for the first cleavage division in mice
Source: Biol Open. 2015 Jan 23;4(2):212–23. doi: 10.1242/bio.201410959 (PMC4365490; doi:10.1242/bio.201410959)
Supplement: Supplementary Material [file supp_4_2_212__index.html]

mir-34b/c and mir-449a/b/c are required for spermatogenesis, but not for the first cleavage division in mice — Supplementary Material 

# mir-34b/c and mir-449a/b/c are required for spermatogenesis, but not for the first cleavage division in mice

## bio.201410959 Supplementary Material

**Files in this Data Supplement:**

- Supplementary Material - Shuiqiao Yuan et al. doi: 10.1242/bio.201410959
- Table S4 - A list of dysregulated genes in miR-dKO round spermatids (p<0.05; upregulated genes are highlighted in red and downregulated ones in yellow).
- Table S5 - 353 target genes of the five miRNAs (miR34b/c and miR-449a/b/c) detected in miR-dKO round spermatids by RNA-Seq (upregulated genes are highlighted in red, and downregulated ones in yellow; genes without changes are labeled with green).
- Table S6 - Changes of 89 target genes of the five miRNAs (miR34b/c and miR-449a/b/c) known to be essential for late spermiogenesis in miR-dKO round spermatids (upregulated genes are highlighted in red, and downregulated ones in yellow; genes without changes are labeled with green).
