# Supplementary material for: mir-34b/c and mir-449a/b/c are required for spermatogenesis, but not for the first cleavage division in mice
Source: Biol Open. 2015 Jan 23;4(2):212–23. doi: 10.1242/bio.201410959 (PMC4365490; doi:10.1242/bio.201410959)
Supplement: Supplementary Material [file supp_bio.201410959_bio.201410959-s1.pdf]

Biology Open (2015) 000, 1–12 doi:10.1242/bio.201410959

Supplementary Material
Shuiqiao Yuan et al. doi: 10.1242/bio.201410959

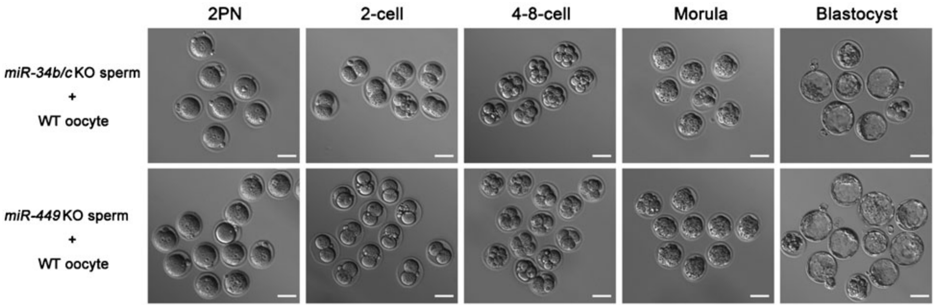

Fig. S1. Morphology of embryos at 2-pronuleus (PN), 2-cell, 4–8-cell, morula and blastocyst stages of development. Sperm and oocyte genotypes are labeled to the left. Scale bars = 100 µm.

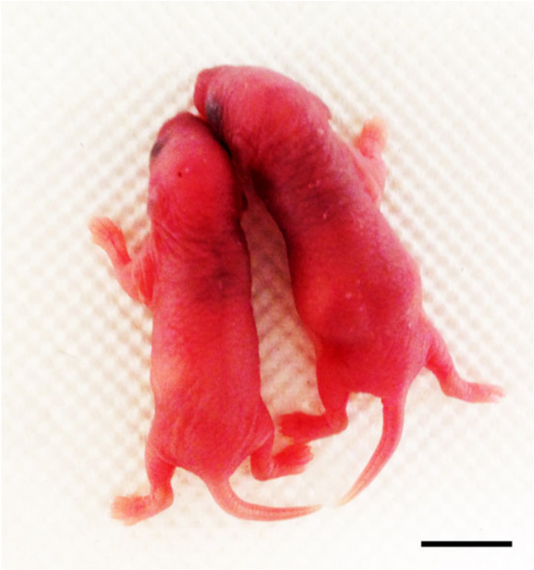

Fig. S2. Pups born through ROSI using WT oocytes and *miR-34b/c* and *miR-449* double knockout round spermatids. Scale bar = 1 cm.

Table S1. Fertility of *miR-34b/c* KO, *miR-449* KO and miR-dKO mice

Male genotype	Female genotype	No. of litters	No. of pups	Average of litter size	Average of litter interval (days)
WT	WT	26	215	8.27 ± 0.66	23.04 ± 0.12
miR-34b/c KO	WT	20	149	7.45 ± 1.05	24.35 ± 0.21
miR-34b/c KO	*miR-34b/c* KO	15	109	7.26 ± 1.33	23.26 ± 0.22
miR-449 KO	WT	18	145	8.06 ± 0.78	24.24 ± 0.34
miR-449 KO	*miR-449* KO	24	170	7.08 ± 1.49	23.82 ± 0.83
miR-dKO	WT	0	0	0	0

Biology Open (2015) 000, 1–12 doi:10.1242/bio.201410959

Table S2. Development of WT oocytes or *miR-34b/c* KO oocytes injected with *miR-34b/c* KO spermatozoa

Oocyte genotype	Total No. of surviving oocytes (no. of exp.)	No. of oocytes with 2PN (% of $\frac{2PN}{Total}$)	No. of 2-cell (% of $\frac{2\text{-cell}}{2PN}$)	No. of 4-cell (% of $\frac{4\text{-cell}}{2\text{-cell}}$)	No. of morula (% of $\frac{morula}{2\text{-cell}}$)	No. of Blastocyst (% of $\frac{Blastocyst}{2\text{-cell}}$)
WT	38 (2)	31 (81.58)	29 (93.55)	28 (96.55)	26 (89.66)	16 (55.17)
miR-34b/c KO	70 (2)	59 (84.29)	52 (88.13)	49 (94.23)	43 (82.69)	28 (53.85)
Parthenogenetic control	30 (2)	3	1	0	0	0

exp., experiment.

Table S3. Development of WT oocytes or *miR-449* KO oocytes injected with *miR-449* KO spermatozoa

Oocyte genotype	Total No. of surviving oocytes (no. of exp.)	No. of oocytes with 2PN (% of $\frac{2PN}{Total}$)	No. of 2-cell (% of $\frac{2\text{-cell}}{2PN}$)	No. of 4-cell (% of $\frac{4\text{-cell}}{2\text{-cell}}$)	No. of morula (% of $\frac{morula}{2\text{-cell}}$)	No. of Blastocyst (% of $\frac{Blastocyst}{2\text{-cell}}$)
WT	124 (3)	108 (87.10)	102 (94.44)	95 (93.14)	87 (85.29)	59 (57.84)
miR-449 KO	137 (4)	116 (84.67)	110 (94.83)	101 (91.82)	91 (82.73)	57 (51.82)
Parthenogenetic control	30 (2)	3	1	0	0	0

exp., experiment.

Biology Open (2015) 000, 1–12 doi:10.1242/bio.201410959

Tables S4–S6: See supplementary webpage

Table S7. Primer sequences for qPCR validation of RNA-Seq data

Gene symbol	Forward (5′→3′)	Reverse (5′→3′)
Tekt3	GTGCAGGGATATGGCTCAGT	TTCACAGCCAAGTCATGCTC
Zpbp2	TTTCCTGGAGCCGTTATCTG	ATCAGGTTCCCGATATGCAA
Adam2	CTTGACCACAGGCGATGCTAA	TGATAGGTAGCGCCAACATAATC
Tekt4	GCAGTGAACTCGGCTTTCTC	TGGTCTGTGATCTCCGTCAG
Nphp1	GATAGTTTGGTGACTGAAAGCCA	CCCACAGGTGCAGCTTCAT
Tekt2	TTGAAGCCACCAAGAAGGTC	CCGGTGGTCAGAGTTGAGTT
Cplx1	AGTTCGTGATGAAACAAGCCC	TCTTCCTCCTTCTTAGCAGCA
Pebp1	GAGCTGGGCAAAGTGCTAAC	GTCTGTGAGGACCAGGGTGT
Adam3	TTGCGTGACAAGTTGGGAACA	GGGCTCAATTCCATAAGTGATGT
Gamt	CACGCACCTGCAAATCCTG	TACCGAAGCCCACTTCCAAGA
Sepp1	AGCCATTAAGATCGCTTACTGTG	GAGGGCTCCGCAGTTTTATTG
Prnd	TCTCCACGGTCAAGGCAAG	CGGTTCTCAGCTACCCGAG
Wipf3	ACACCCCCAGCTTAAGGAAG	CCTTTTGAACCCTCGATCTG
Mfge8	ATCTACTGCCTCTGCCCTGA	CCAGACATTTGGCATCATTG
Gapdh	AACTTTGGCATTGTGGAAGG	ACACATTGGGGGTAGGAACA
